# T-Type Calcium Channels Contribute to Burst Firing in a Subpopulation of Medial Habenula Neurons

**DOI:** 10.1523/ENEURO.0201-20.2020

**Published:** 2020-08-11

**Authors:** Casey R. Vickstrom, Xiaojie Liu, Yuqi Zhang, Lianwei Mu, Thomas J. Kelly, Xudong Yan, Meng-ming Hu, Shana T. Snarrenberg, Qing-song Liu

**Affiliations:** Department of Pharmacology and Toxicology, Medical College of Wisconsin, Milwaukee, WI 53226

**Keywords:** burst, calcium, habenula, T-type channel

## Abstract

Action potential (AP) burst firing caused by the activation of low-voltage-activated T-type Ca^2+^ channels is a unique mode of neuronal firing. T-type channels have been implicated in diverse physiological and pathophysiological processes, including epilepsy, autism, and mood regulation, but the brain structures involved remain incompletely understood. The medial habenula (MHb) is an epithalamic structure implicated in anxiety-like and withdrawal behavior. Previous studies have shown that MHb neurons fire tonic APs at a frequency of ∼2–10 Hz or display depolarized low-amplitude membrane oscillations. Here, we report in C57BL/6J mice that a subpopulation of MHb neurons are capable of firing transient, high-frequency AP bursts mediated by T-type channels. Burst firing was observed following rebounding from hyperpolarizing current injections or during depolarization from hyperpolarized membrane potentials in ∼20% of MHb neurons. It was rarely observed at baseline but could be evoked in MHb neurons displaying different initial activity states. Further, we show that T-type channel mRNA, in particular Ca_v_3.1, is expressed in the MHb in both cholinergic and substance P-ergic neurons. Pharmacological Ca_v_3 antagonism blocked both burst firing and evoked Ca^2+^ currents in MHb neurons. Additionally, we observed high-frequency AP doublet firing at sustained depolarized membrane potentials that was independent of T-type channels. Thus, there is a greater diversity of AP firing patterns in MHb neurons than previously identified, including T-type channel-mediated burst firing, which may uniquely contribute to behaviors with relevance to neuropsychiatric disease.

## Significance Statement

Previous studies have reported that medial habenula (MHb) neurons solely fire tonic action potentials (APs) at ∼2–10 Hz or display depolarized low amplitude membrane oscillations. In contrast, we found that a subpopulation of MHb neurons fire high-frequency AP bursts that share the characteristics of T-type Ca^2+^ channel-mediated low threshold spikes and were blocked by pharmacological antagonism of T-type Ca^2+^ channels. T-type Ca^2+^ channel mRNA, especially Ca_v_3.1, is expressed in the MHb in both cholinergic and substance P-ergic neurons. T-type channel-independent AP doublets were also observed in MHb neurons at sustained depolarized membrane potentials. Thus, there is a greater diversity of AP firing patterns in MHb neurons than previously recognized, including T-type Ca^2+^ channel-mediated burst firing.

## Introduction

Burst action potential (AP) firing mediated by the activation of low-voltage-activated T-type Ca^2+^ channels is a unique mode of neuronal activity that occurs in various neuronal populations, particularly in thalamic, septal, and sensory neurons (Perez-Reyes, 2003; Cheong and Shin, 2013; Lambert et al., 2014). This burst firing has been shown to mediate important physiological and pathophysiological processes, including the synchronization of the thalamocortical circuit and the generation of rhythmic neuronal activity during normal sleep (Huguenard and McCormick, 2007), spike-wave discharges in absence epilepsy (Huguenard and McCormick, 2007), and neuropathic pain (Bourinet et al., 2016). Additionally, mutations in T-type Ca^2+^ channel genes have been implicated in epilepsy, autism spectrum disorder, and schizophrenia (Weiss and Zamponi, 2020). There are three distinct T-type Ca^2+^ channel genes: *CACNA1G*, *CACNA1H*, and *CACNA1I*, which encode the pore-forming α1 subunits known as Ca_v_3.1, Ca_v_3.2, and Ca_v_3.3, respectively (Perez-Reyes, 2003). In contrast to the other voltage-gated Ca^2+^ channels, T-type Ca^2+^ channels have unique features, including low-voltage activation, rapid voltage-dependent inactivation, and the generation of transient currents that can trigger a brief burst of high-frequency APs (Llinás and Jahnsen, 1982; Perez-Reyes, 2003; Cheong and Shin, 2013). As a large proportion of T-type Ca^2+^ channels are inactivated at typical resting membrane potentials (*V*_m_), neuron hyperpolarization can de-inactivate T-type Ca^2+^ channels and trigger a burst of APs, a phenomenon called “rebound bursting” (Llinás and Jahnsen, 1982; Perez-Reyes, 2003; Cheong and Shin, 2013). Burst firing can have unique functional consequences, including information encoding by enhancing signal-to-noise ratio, facilitation of neuropeptide release, and increasing the reliability of synaptic transmission (Lisman, 1997; Schultz et al., 1997; van den Pol, 2012).

The habenula is a phylogenetically conserved epithalamic brain structure that is broadly classified into medial [medial habenula (MHb)] and lateral (LHb) subnuclei (Sutherland, 1982; Antolin-Fontes et al., 2015; Boulos et al., 2017). The MHb regulates anxiety-like and depressive-like behavior (Görlich et al., 2013; Hsu et al., 2014, 2016), fear extinction (Soria-Gómez et al., 2015; Zhang et al., 2016), nicotine intake and withdrawal (Fowler et al., 2011; Frahm et al., 2015; Zhao-Shea et al., 2015; Tuesta et al., 2017), opioid withdrawal (Boulos et al., 2020), and stress responsivity in zebrafish (Agetsuma et al., 2010; Lee et al., 2010; Mathuru and Jesuthasan, 2013). There is well-documented evidence that neurons of the LHb are capable of firing bursts of APs mediated by T-type Ca^2+^ channel activation (Wilcox et al., 1988; Chang and Kim, 2004; Cui et al., 2018; Yang et al., 2018). Recent work has shown that LHb burst firing contributes to the development of depressive-like behavior in rodents and that the rapid antidepressant effects of ketamine are mediated by blockade of LHb burst firing (Cui et al., 2018; Yang et al., 2018). In contrast, previous electrophysiological studies report that MHb neurons solely fire tonic, regular APs at a frequency between ∼2 and 10 Hz (Kim and Chang, 2005; Kim and Chung, 2007; Görlich et al., 2013; Sakhi et al., 2014; Shih et al., 2014; Choi et al., 2016; Otsu et al., 2018; Weiss et al., 2018; Arvin et al., 2019; Ge et al., 2019).

In this study, we identified a subpopulation of MHb neurons that are capable of firing high-frequency AP bursts. Although only ∼7% of MHb neurons displayed burst firing at baseline, ∼20% displayed burst firing following membrane hyperpolarization or with depolarization from a hyperpolarized *V*_m_. Burst firing from a hyperpolarized *V*_m_ was blocked by the selective T-type Ca^2+^ channel antagonist Z944 (Tringham et al., 2012). We identified robust expression of Ca_v_3.1 mRNA predominantly in the lateral MHb using RNAscope *in situ* hybridization, which co-localized with tachykinin-1 (Tac1) and choline acetyltransferase (ChAT), markers which define neurons of the dorsal and ventral MHb, respectively. These neurons have distinct anatomic projections to the interpeduncular nucleus and have unique roles in regulating anxiety-like and depressive-like behavior and nicotine dependence (Molas et al., 2017). Thus, MHb neuron burst firing, especially that mediated by T-type channel activation, is a previously unidentified mode of neuronal activity that could regulate diverse behaviors relevant to neuropsychiatric disorders.

## Materials and Methods

### Animals

All animal procedures were performed in accordance with the Medical College of Wisconsin animal care committee’s regulations. C57BL/6J mice were given *ad libitum* access to food and water and housed four to five per cage in a temperature (23 ± 1°C) and humidity-controlled room (40–60%) with a 14/10 h light/dark cycle. All experiments were performed on adult male or female C57BL/6J mice (8–10 weeks old). C57BL/6J mice were obtained from The Jackson Laboratory. Experiments were performed between zeitgeber time (ZT)8 and ZT14, where ZT0 is lights on.

### Slice preparation and electrophysiology

Mice were anesthetized by isoflurane inhalation and decapitated. The brain was trimmed and embedded in 3% low-melting-point agarose, and coronal slices containing the MHb (200 μm thick) were cut using a vibrating slicer (Leica VT1200s). Slices were prepared in a NMDG-based solution containing the following: 92 mm NMDG, 2.5 mm KCl, 1.25 mm NaH_2_PO_4_, 26 mm NaHCO_3_, 20 mm HEPES, 25 mm glucose, 2 mm thiourea, 5 mm Na-ascorbate, 3 mm Na-pyruvate, 0.5 mm CaCl_2_·2H_2_O, and 7 mm MgSO_4_ (pH 7.3–7.4 with HCl). Artificial CSF (ACSF), containing the following: 119 mm NaCl, 3 mm KCl, 2 mm CaCl_2_, 1 mm MgCl_2_, 1.25 mm NaH_2_PO_4_, 25 mm NaHCO_3_, and 10 mm glucose, was gradually spiked-in to the NMDG-containing solution after slice cutting in 5-min intervals for a total of 20 min at room temperature, similar to a previously described approach (Ting et al., 2018). Some slices were cut in a sucrose-based solution containing the following: 68 mm sucrose, 78 mm NaCl, 3 mm KCl, 2 mm CaCl_2_, 1 mm MgCl_2_, 1.25 mm NaH_2_PO_4_, 25 mm NaHCO_3_, and 25 mm glucose. These slices were incubated in the same solution for 20 min after cutting. The slices were then allowed to recover for at least 1 h in ACSF. All solutions were saturated with 95% O_2_ and 5% CO_2_.

Whole-cell patch-clamp recordings were made using patch-clamp amplifiers (Multiclamp 700B) under infrared differential interference contrast (DIC) microscopy. Data acquisition and analysis were performed using DigiData 1550B digitizers and the analysis software pClamp 10.7 (Molecular Devices). Signals were filtered at 2 kHz and sampled at 10 kHz. Recordings were performed in the presence of the AMPA receptor antagonist 6-cyano-7-nitroquinoxaline-2,3-dione disodium salt (CNQX; 10 μm) and GABA_A_ receptor antagonist picrotoxin (50 μm) unless otherwise stated. For before-and-after comparisons, the selective T-type Ca^2+^ channel antagonist Z944 (3 μm), the NMDA receptor antagonists (*RS*)−3-(2-carboxypiperazin-4-yl)-propyl-1-phosphonic acid (CPP; 5 μm) or *D*-(-)−2-amino-5-phosphonopentanoic acid (AP-5; 50 μm), or CNQX (20 μm) were bath perfused in ACSF in recordings where neurons fired apparent bursts, and current protocols that induced bursting before drug perfusion were repeated after drug perfusion. Ca^2+^ current recordings used two different voltage protocols to evoked Ca^2+^ currents. For determining the voltage dependence of activation, neurons were first held for 1 s at −90 mV, followed by a 320-ms step to progressively more depolarized holding potentials in 5-mV increments, beginning at −90 mV and finishing at −30 mV, followed by return to −90 mV holding for 500 ms. Neurons were held at −70 mV between sweeps. For determining the voltage dependence of inactivation, neurons were first held for 3.6 s at progressively more depolarized membrane potentials in 5-mV increments, beginning at −120 mV and finishing at −50 mV. After this initial holding potential, neurons were stepped to −50 mV for 320, followed by holding at −90 mV for 1 s. Neurons were held at −70 mV between sweeps. Glass pipettes (3–6 MΩ) for current clamp recordings were filled with an internal solution containing the following: 140 mm K-gluconate, 5 mm KCl, 10 mm HEPES, 0.2 mm EGTA, 2 mm MgCl_2_, 4 mm Mg-ATP, 0.3 mm Na_2_GTP, and 10 mm Na_2_-phosphocreatine (pH 7.2 with KOH). Glass pipettes for voltage clamp recordings of Ca^2+^ currents were filled with an internal solution containing the following: 135 mm tetramethyl ammonium (TMA)-OH, 10 mm EGTA, 2 mm MgCl_2_, and 40 mm HEPES, titrated to pH 7.2 with hydrofluoric acid (HF; Todorovic and Lingle, 1998). Series resistance (15–30 MΩ) was monitored throughout all recordings, and data were discarded if the resistance changed by >20%. Liquid junction potentials were not corrected for. All recordings were performed at 32 ± 1°C using an automatic temperature controller (Warner Instruments).

### RNAscope *in situ* hybridization

Mice were deeply anesthetized with isoflurane and transcardially perfused with 0.1 m sodium PBS followed by 4% paraformaldehyde in 4% sucrose-PBS (pH 7.4). After perfusion, the brain was removed and postfixed in the same fixative for 4 h at 4°C and was then dehydrated in increasing concentrations of sucrose (20% and 30%) in 0.1 m PBS at 4°C and frozen on dry ice. Coronal MHb sections (10 μm) were cut with a Leica cryostat and mounted on Superfrost Plus microscope slides (Fisher Scientific). Probes targeting the mRNA transcripts of Ca_v_3.1 (target region: base pairs 1263–2886 of *Mus musculus* CACNA1G, NM_009783.3), Ca_v_3.2 (target region: base pairs 2287–4243 of *M. musculus* CACNA1H, NM_021415.4), Ca_v_3.3 (target region: base pairs 1259–2600 of *M. musculus* CACNA1I, NM_001044308.2), ChAT (target region: base pairs 1090–1952 of *M. musculus* ChAT, NM_009891.2), and Tac1 (target region: base pairs 20–1034 of *M. musculus* tachykinin 1, NM_009311.2) were designed by and purchased from Advanced Cell Diagnostics Inc. The experiment was conducted as per the manufacturer’s instructions for the RNAscope Multiplex Fluorescent V2 Assay. Stained slides were mounted with ProLong Gold Antifade Mountant with DAPI (Invitrogen) and imaged on a Leica TCS SP8 confocal microscope. A probe targeting *Bacilus subtilis* protein DapB was used as a negative control, whereas probes targeting the ubiquitously expressed Polr2a, PPIB, and UBC served as positive controls.

### Statistics and data analysis

Paired *t* tests were used to analyze bursting before and after drug perfusion in current clamp recordings. Two-way ANOVA with repeated measures was used to analyze the effect of Z944 on evoked Ca^2+^ currents. Expression of T-type channel mRNA was analyzed by one-way ANOVA on ranks for Ca_v_3.1, Ca_v_3.2, and Ca_v_3.3 from MHb slices because of non-normally distributed data (Shapiro–Wilk *p *<* *0.05), followed by Dunn’s *post hoc* multiple comparison testing. Ca_v_3.1 expression in the medial versus lateral MHb was analyzed by unpaired *t* test. The voltage-dependencies of activation and inactivation were described by the following Boltzmann functions:

$$\text{Activation}:G\left(V\right)\;=\;G_{\text{max}}/\left(\text{1}\;+\;\text{exp}\;\left[-\;\left(V-V_{\text{5}0}\right)/\text{k}\right]\right)$$

$$\text{Inactivation}:I\left(V\right)\;=\;I_{\text{max}}/\left(\text{1}\;+\;\text{exp}\left[\left(V-V_{\text{5}0}\right)/k\right]\right)$$

## Results

### Diverse electrophysiological states of MHb neurons

**Figure 1.**
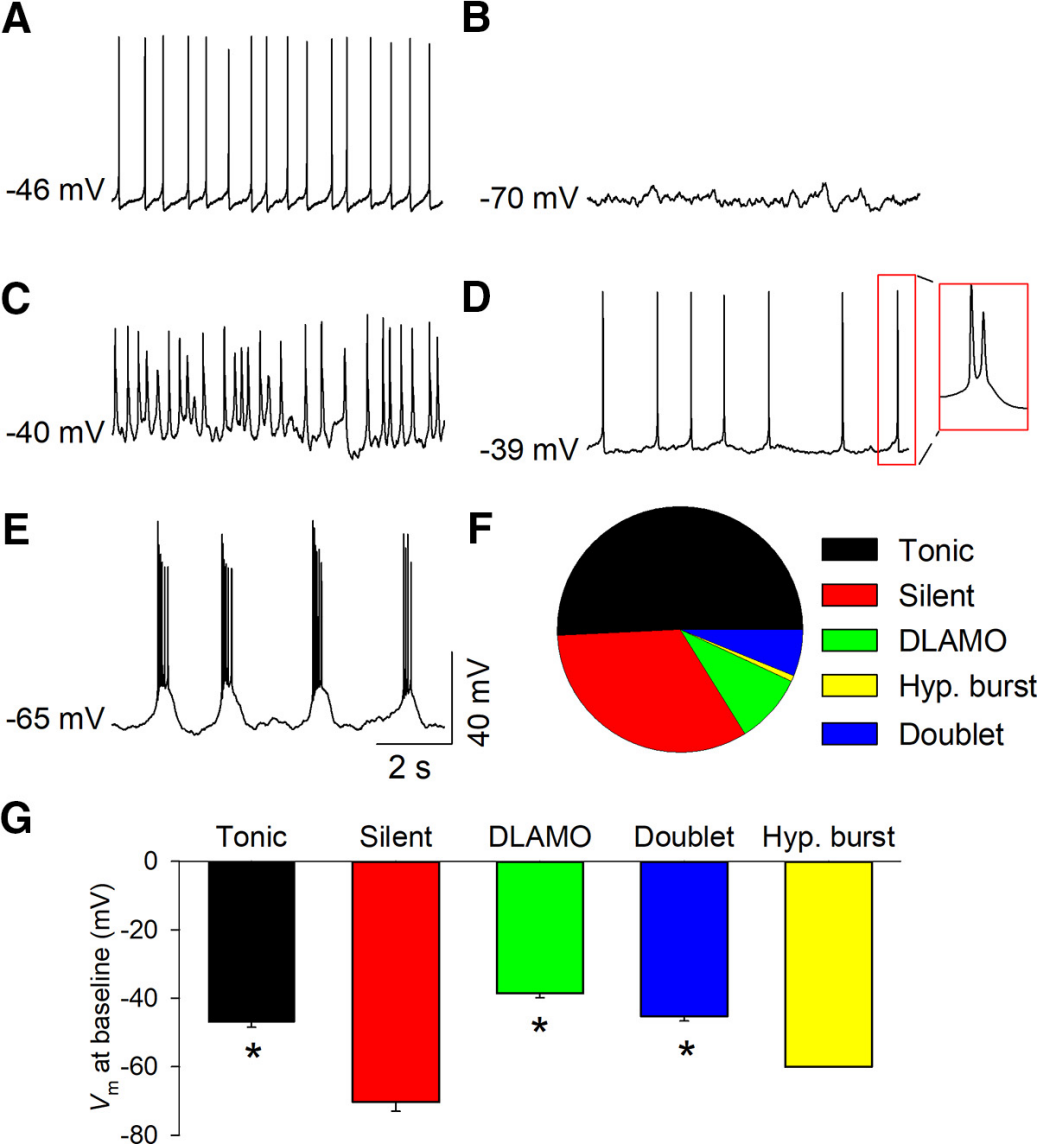
Diverse activity states of MHb neurons. MHb neurons displayed diverse activity states at baseline. The majority of neurons exhibited tonic AP firing (***A***), whereas others were silent (***B***), displayed DLAMOs (***C***), fired AP doublets at depolarized membrane potentials (***D***), or bursted at a hyperpolarized membrane potential (***E***). ***F***, Percent of MHb neurons that displayed different activity states at baseline. ***G***, Average resting membrane potential of different cell states; *p <* *0.05 versus silent. Hyp., hyperpolarized.

Cell-attached and whole-cell patch-clamp recordings were made to determine the activity state of MHb neurons at baseline. In whole-cell recordings, an assessment was made in the first few seconds to avoid alteration of cell state by the internal solution. MHb neurons exhibited a variety of distinct activity states at baseline. About half (66 of 130, 50.8%) of MHb neurons exhibited spontaneous, tonic AP firing at a frequency of ∼2–10 Hz (Fig. 1*A*,*F*). About a third (43 of 130, 33.1%) were electrophysiologically silent (Fig. 1*B*,*F*). A minority (12 of 130, 9.2%) of MHb neurons exhibited depolarized low-amplitude membrane oscillations (DLAMOs; Fig. 1*C*,*F*), characterized by oscillatory fluctuations in membrane potential (*V*_m_; Sakhi et al., 2014). Additionally, we observed a small population of neurons that exhibited high-frequency AP firing. At baseline, most spontaneously high-frequency AP firing MHb neurons fired AP doublets at a frequency of ∼75–125 Hz at an average resting *V*_m_ of −45.2 ± 1.5 mV (Fig. 1*D*; 8 of 130 total neurons, 6.2%; Fig. 1*F*), whereas one neuron spontaneously fired long trains of APs on top of a prominent depolarized plateau potential followed by an afterdepolarization (Fig. 1*E*; 1 of 130 total neurons, 0.8%; Fig. 1*F*). The average intraburst frequency was 27.2 ± 1.8 Hz, and the average interburst interval was 2.3 ± 0.6 s in this neuron. The average resting *V*_m_ in silent cells was significantly more hyperpolarized relative to that in tonic, silent, DLAMO, and depolarized doublet firing MHb neurons (Fig. 1*G*). Thus, MHb neurons display a variety of electrophysiological states at baseline, including high-frequency AP firing that has not been previously reported.

### Burst firing from hyperpolarized membrane potentials

Although burst firing in MHb neurons has not been previously reported, neurons of the nearby LHb and thalamus display burst firing that resembles the hyperpolarized burst firing we observed, which is mediated by the activation of T-type Ca^2+^ channels (Wilcox et al., 1988; Perez-Reyes, 2003; Chang and Kim, 2004; Cheong and Shin, 2013; Lambert et al., 2014; Cui et al., 2018; Yang et al., 2018). T-type channels are a member of the low-voltage activated (LVA) family of voltage-gated Ca^2+^ channels (Perez-Reyes, 2003; Cheong and Shin, 2013). They can be activated by low-level membrane depolarization, though sustained depolarization leads to channel inactivation and can cause a switch to tonic firing (McCormick and Pape, 1990). We thus tested whether depolarization of MHb neurons from a hyperpolarized *V*_m_ can trigger burst firing and whether prolonged depolarization causes a transition to tonic firing.

**Figure 2.**
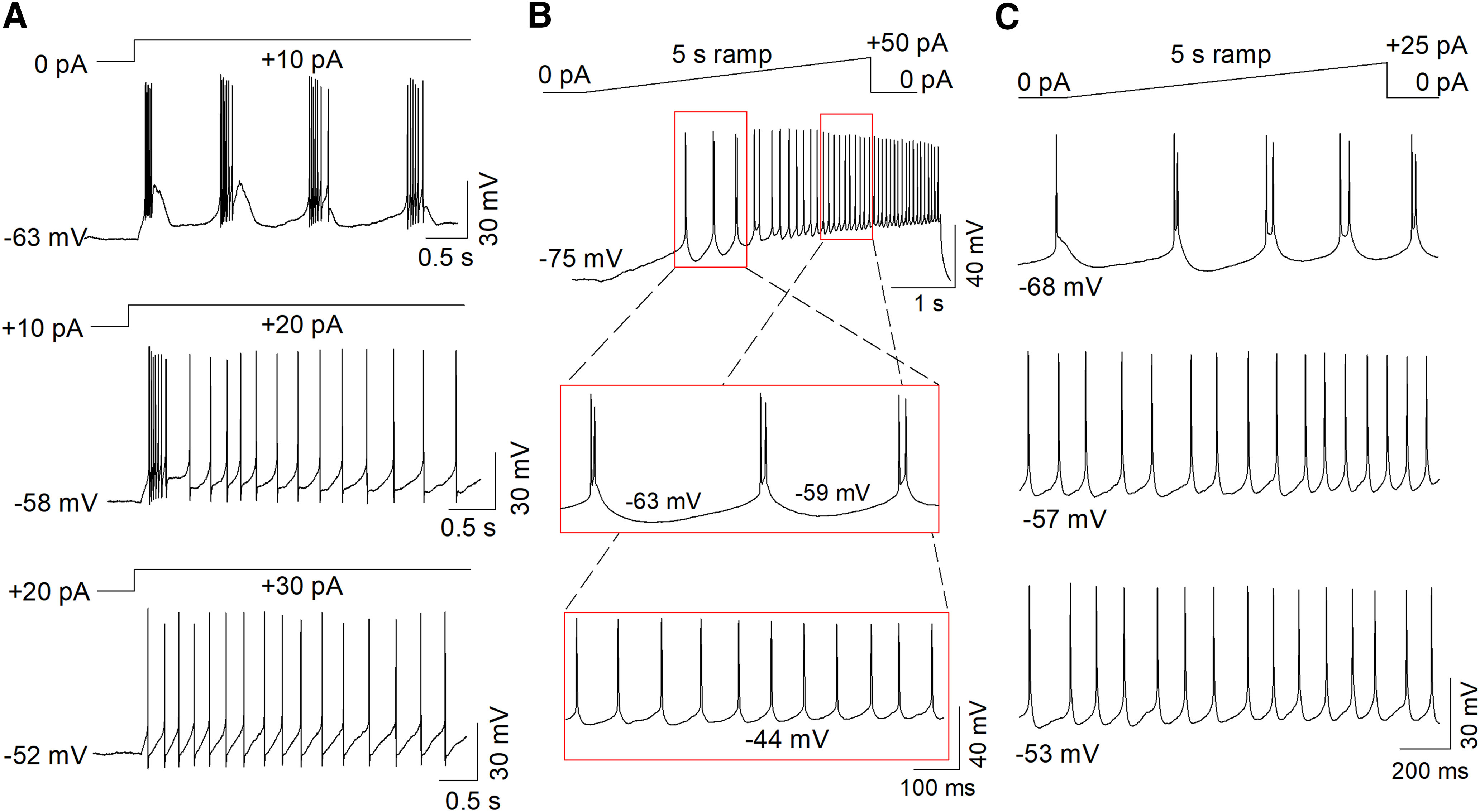
Depolarization from hyperpolarized membrane potentials triggered burst firing in a subset of MHb neurons. ***A***, Step depolarization triggered burst firing from a resting *V*_m_ of −63 mV. With constant current injection to a *V*_m_ of −58 mV, step depolarization triggered initial burst firing followed by tonic firing. With constant current injection to a *V*_m_ of −52 mV, step depolarization triggered solely tonic firing. ***B***, Ramp depolarization from a *V*_m_ <−60 mV triggered initial burst firing in 30 of 152 MHb neurons (19.7%). Tonic firing was observed as the *V*_m_ depolarized above approximately −55 mV. ***C***, Bursting was observed when the *V*_m_ was −68 mV before ramp depolarization but was abolished with constant current injection to −57 or −53 mV before the ramp.

In a silent MHb neuron, a depolarization step (10 pA) from the resting *V*_m_ (−63 mV) triggered high-frequency AP firing (AP frequency = 85 Hz) that resembled the hyperpolarized burst firing observed in Figure 1*E*, in that it fired a train of APs on top of a depolarized plateau potential and was followed by a large afterdepolarization (Fig. 2*A*). When the *V*_m_ was changed to −58 mV through constant current injection (10 pA), the same depolarization step (10 pA) triggered transient burst firing at 34 Hz, followed by tonic firing at 3.5 Hz. When the *V*_m_ was further depolarized to −52 mV by 20-pA constant current injection, the 10-pA depolarization step triggered tonic firing at 3.8 Hz. Similarly, ramp depolarization (0 to +50 pA ramp, 5-s duration) from a resting *V*_m_ of −75 mV triggered initial burst firing in an MHb neuron (Fig. 2*B*). As the *V*_m_ became more depolarized during the ramp, burst firing transitioned to tonic firing. Burst firing remained when the *V*_m_ was more hyperpolarized than approximately −55 mV but transitioned to tonic firing as the *V*_m_ depolarized above this level. At a *V*_m_ of −44 mV, only repetitive tonic firing was observed. In a different MHb neuron, initial burst firing to ramp depolarization was similarly observed from a resting *V*_m_ of −68 mV, but burst firing was not observed with constant current injection to −57 or −53 mV before the ramp (Fig. 2*C*). Ramp depolarization-induced burst firing from a hyperpolarized *V*_m_ (<−60 mV before ramp) triggered initial burst firing in 30 of 152 MHb neurons (19.7%). Burst firing from a hyperpolarized *V*_m_ that is abolished by holding the neuron at depolarized potentials suggests that T-type channel activation contributes to the initial burst firing.

**Figure 3.**
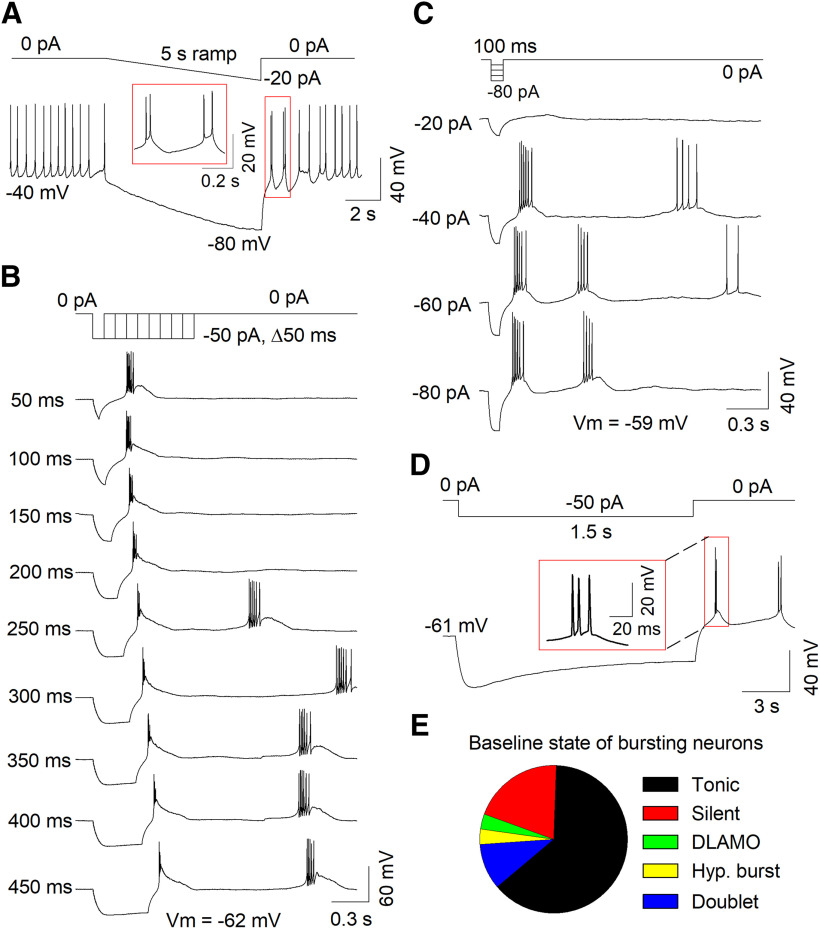
Hyperpolarization triggered burst firing in a subset of MHb neurons. ***A***, Ramp hyperpolarization in a tonic firing neuron with a resting *V*_m_ of −40 mV converted tonic firing to burst firing. ***B***, Progressively longer hyperpolarizing current injections triggered rebound burst firing in an MHb neuron. ***C***, Progressively greater hyperpolarizing current injections triggered rebound burst firing in an MHb neuron. ***D***, A 1.5-s-long hyperpolarizing current injection triggered rebound burst firing in an MHb neuron. ***E***, Baseline state of MHb neurons that displayed burst firing from a hyperpolarized *V*_m_ (<−60 mV).

T-type channels are nearly completely inactivated when neurons are held at a *V*_m_ >−60 mV (Perez-Reyes, 2003; Cheong and Shin, 2013). As the average *V*_m_ of tonic neurons was −47.0 ± 1.5 mV, it is possible that membrane hyperpolarization may convert these neurons to burst firing neurons. In a tonic firing cell with a resting *V*_m_ of −40 mV, ramp hyperpolarization (0 to −20 pA, 5 s) converted tonic firing to burst firing (Fig. 3*A*). Tonic firing resumed after the *V*_m_ spontaneously depolarized above −50 mV. Thus, MHb neuron firing states can be interchangeable depending on their *V*_m_.

We evaluated whether the offset of a prolonged hyperpolarizing current step could induce rebound burst firing. A series of hyperpolarizing current steps (0 to −50 pA, Δ50 ms) triggered rebound burst firing in an MHb neuron (Fig. 3*B*). Interestingly, as the hyperpolarization duration lengthened, the high-frequency rebound burst was converted to a single AP with a pronounced afterdepolarization, subsequently followed by a spontaneous, high-frequency AP burst. Similarly, progressively greater hyperpolarizing current steps (0 to −80 pA, Δ20 pA, 100 ms) triggered rebound burst firing (Fig. 3*C*). Rebound burst firing following the offset of a prolonged hyperpolarizing current step (≥1 s, −50 pA) was observed in 19.3% (29 of 150) neurons recorded (Fig. 3*D*). Burst firing from a hyperpolarized *V*_m_ could be observed in MHb neurons from all baseline activity states but was most commonly observed in tonic firing neurons (Fig. 3*E*). Thus, depolarization from a hyperpolarized *V*_m_, or hyperpolarization alone, can trigger burst firing in ∼20% of MHb neurons.

### T-type channel expression in the MHb

**Figure 4.**
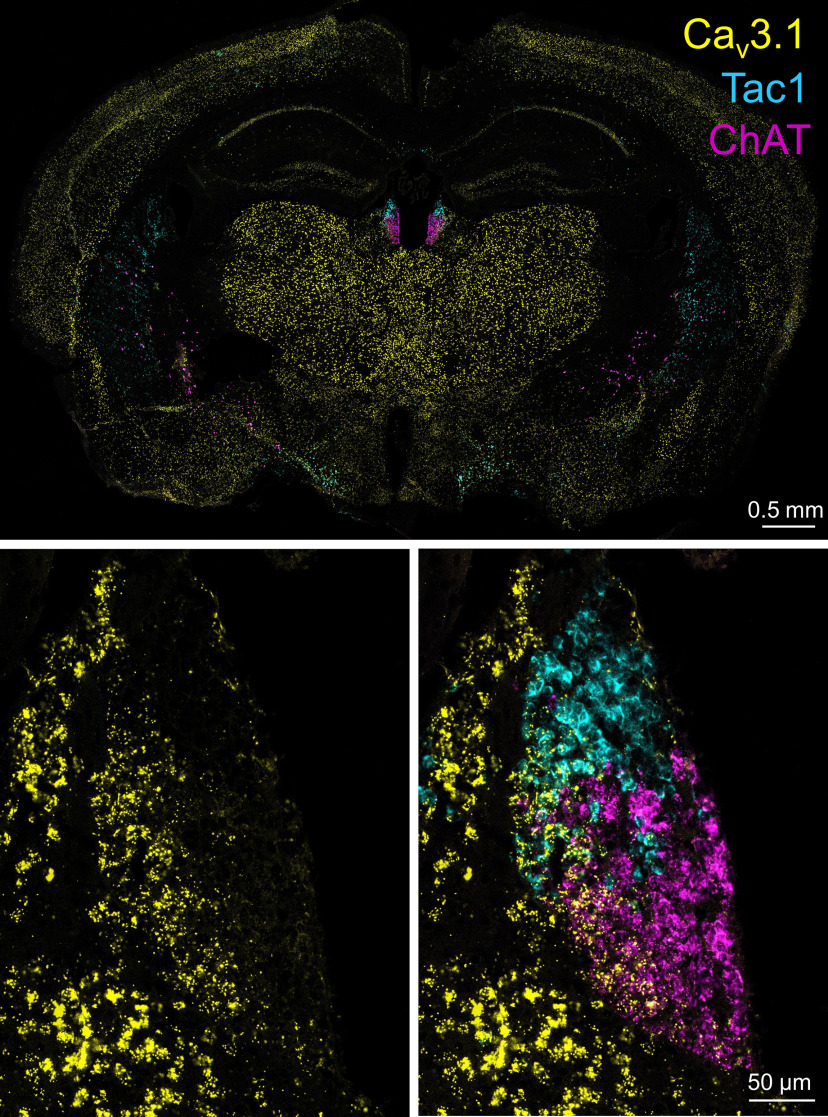
Ca_v_3.1 is expressed in the MHb. RNAscope *in situ* hybridization demonstrated that mRNA for Ca_v_3.1 is expressed in the MHb, in particular its lateral aspect. Both Tac1+ and ChAT+ neurons express Ca_v_3.1 mRNA.

**Figure 5.**
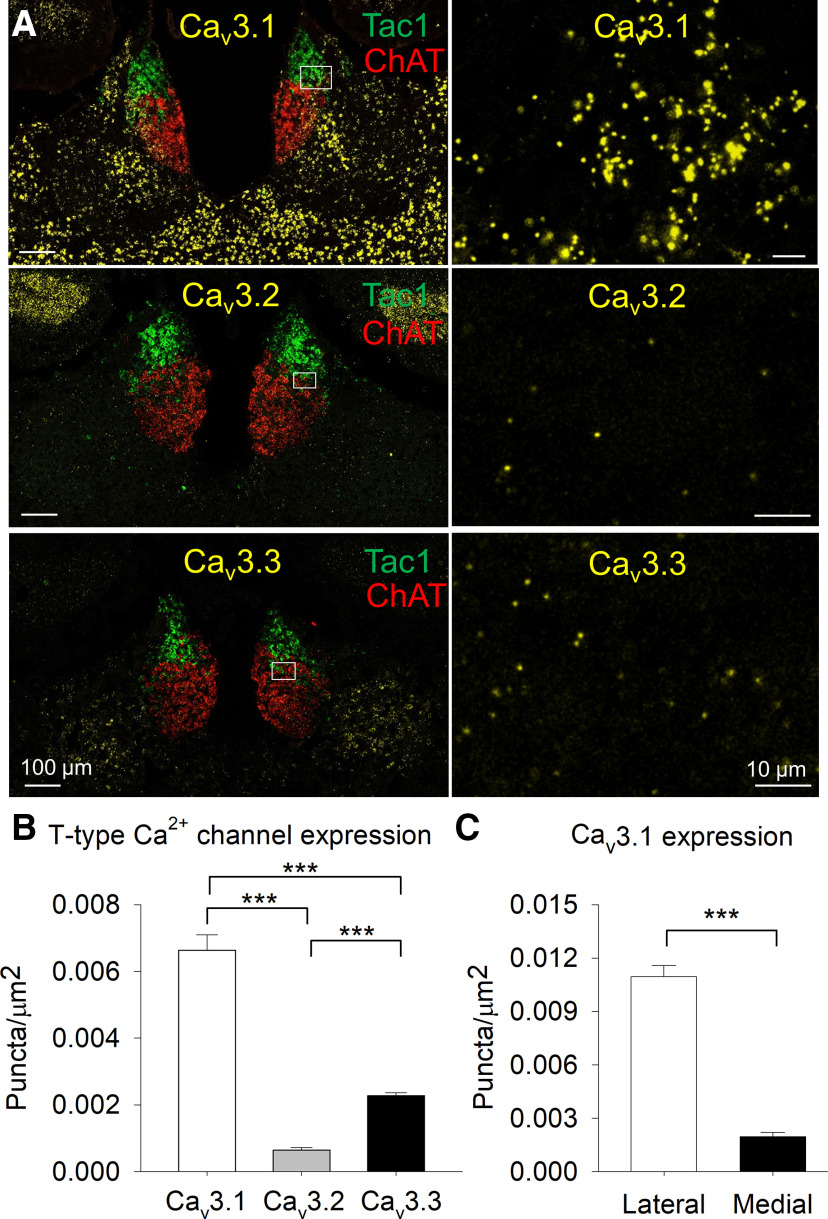
T-type channel expression in the MHb. ***A***, RNAscope *in situ* hybridization for Ca_v_3.1, Ca_v_3.2, and Ca_v_3.3 mRNA in the MHb with Tac1 and ChAT. ***B***, Expression of Ca_v_3.1 is significantly greater than Ca_v_3.2 and Ca_v_3.3 expression, and Ca_v_3.3 expression is significantly greater than Ca_v_3.2 in the MHb; ***p <* *0.001. ***C***, Ca_v_3.1 expression is significantly greater in the lateral MHb than the medial MHb; ***p <* *0.001.

T-type channels are encoded by three genes: *CACNA1G*, *CACNA1H*, and *CACNA1I*, which encode the pore-forming α1 subunits Ca_v_3.1, Ca_v_3.2, and Ca_v_3.3 (Perez-Reyes, 2003). We used RNAscope fluorescent *in situ* hybridization (Wang et al., 2012) to determine whether mRNA for T-type channels is expressed in the MHb and to determine the cell populations that express these channels. We found that mRNA for Ca_v_3.1 was abundantly expressed in the MHb, including in both Tac1+ and ChAT+ neurons (Fig. 4). Expression of Ca_v_3.2 and Ca_v_3.3 was also observed in the MHb in both Tac1+ and ChAT+ neurons, albeit at significantly lower levels than Ca_v_3.1 (Fig. 5*A*,*B*). Ca_v_3.1 expression was significantly greater in the lateral MHb relative to the medial MHb (Fig. 5*C*). Thus, mRNA for T-type channels, in particular Ca_v_3.1, are expressed in the MHb in both major neuron populations.

### Dependence of bursting on T-type channels

**Figure 6.**
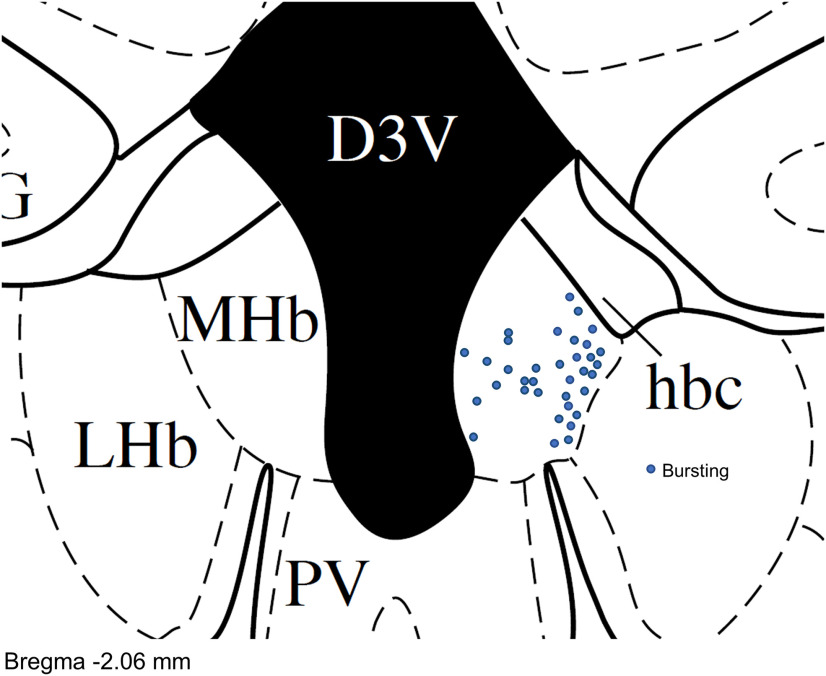
Location of neurons that bursted from a hyperpolarized membrane potential. Neurons that bursted from a *V*_m_ <−60 mV were predominantly located in the lateral and central MHb. Map includes neurons that bursted to ramp depolarization and/or to step hyperpolarization.

**Figure 7.**
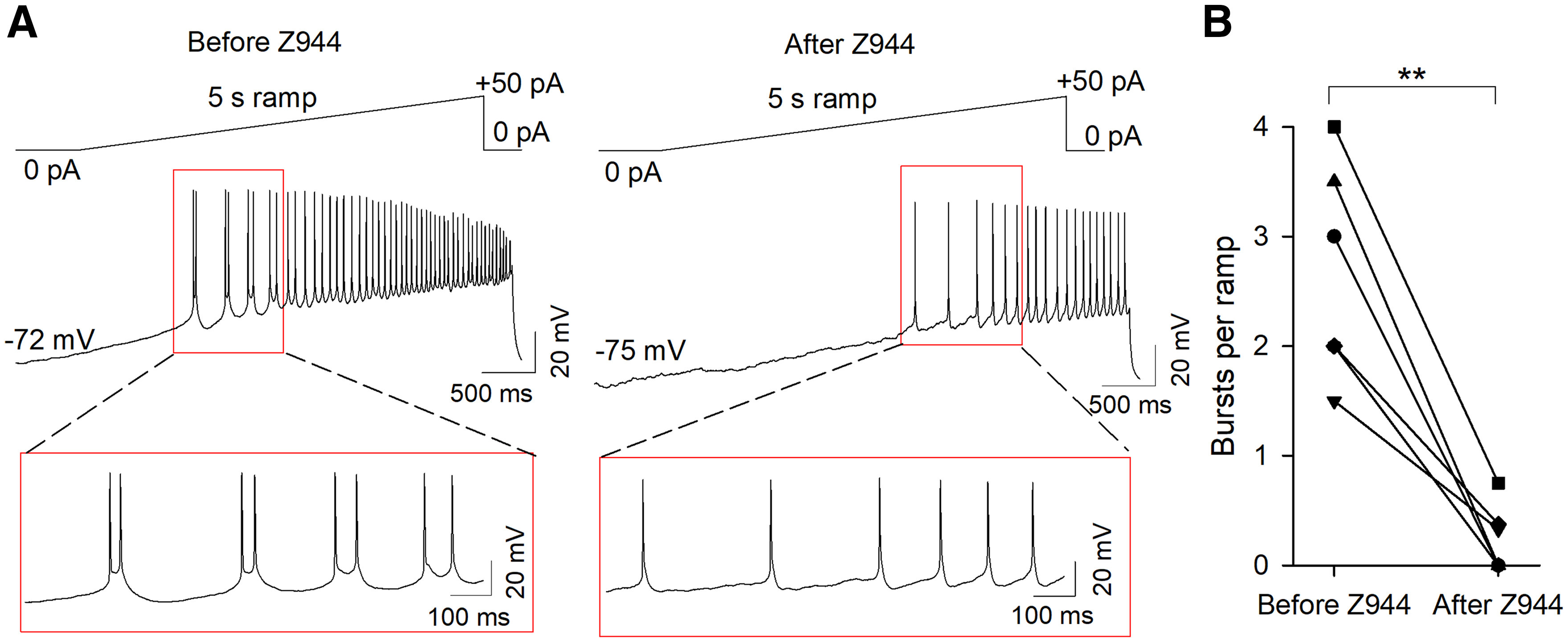
The selective T-type channel antagonist Z944 blocked ramp depolarization-induced burst firing from hyperpolarized membrane potentials. ***A***, Representative traces demonstrating the effect of Z944 to block ramp-induced burst firing from a hyperpolarized *V*_m_. ***B***, The number of bursts per ramp was significantly reduced following Z944 perfusion; **p <* *0.01.

**Figure 8.**
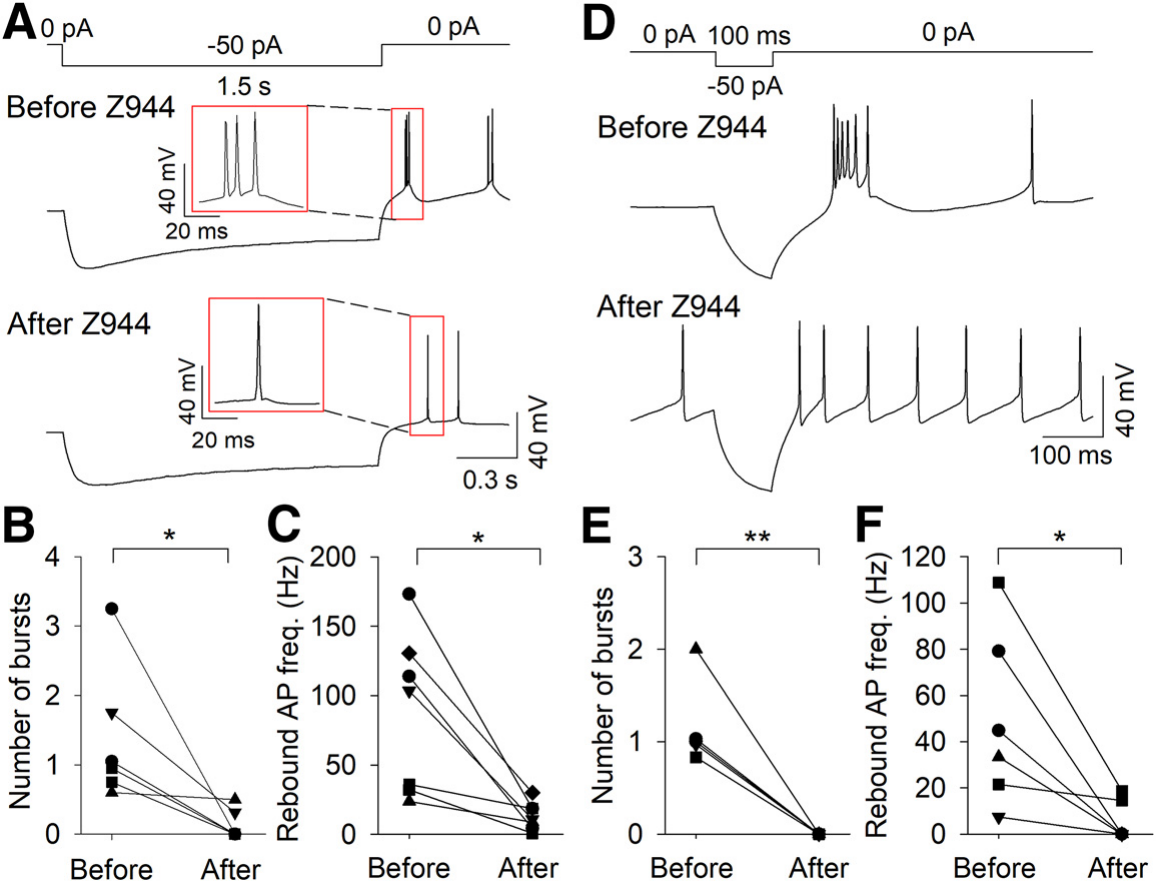
The selective T-type channel antagonist Z944 blocked rebound burst firing. Representative traces demonstrating the effect of Z944 to block rebound burst firing following 1.5-s (***A***) or 100-ms (***D***) hyperpolarization. The number of bursts and rebound AP frequency were significantly reduced following 1.5-s (***B, C***) or 100-ms (***E, F***) hyperpolarization after Z944 perfusion; *p <* *0.05, **p <* *0.01.

We recorded the locations of MHb neurons that displayed high-frequency AP firing from a hyperpolarized *V*_m_. Of the 35 neurons where location information was recorded, 27 of 35 (77.1%) of these neurons were in the lateral or central MHb (Fig. 6), consistent with the highest Ca_v_3 expression in this area. We therefore tested whether pharmacological blockade of T-type channels affected burst firing in MHb neurons in this region. MHb neurons that exhibited ramp depolarization-induced burst firing from a *V*_m_ <−60 mV were bath-perfused with the selective T-type channel antagonist Z944 (3 μm). Z944 significantly reduced the number of bursts per ramp (before Z944: 2.7 ± 0.4, after Z944: 0.2 ± 0.1; *t*_(5)_ = 6.2, *p *=* *0.002, *n* = 6; Fig. 7). Further, Z944 abolished burst firing in a neuron that bursted with ramp depolarization from a *V*_m_ of −75 mV but not from a *V*_m_ of −54 mV (data not shown). MHb neurons that exhibited rebound bursting were bath-perfused with Z944 (Fig. 8). With 1.5-s hyperpolarizing current pulses, Z944 significantly reduced the number of rebound bursts (before Z944: 1.4 ± 0.4, after Z944: 0.1 ± 0.1; *t*_(5)_ = 2.9, *p *=* *0.035, *n* = 6) and rebound AP frequency (before Z944: 87.6 ± 21.8 Hz, after Z944: 13.2 ± 3.8 Hz; *t*_(6)_ = 3.7, *p *=* *0.010, *n* = 7; Fig. 8*A–C*). With 100-ms hyperpolarizing current pulses, Z944 significantly reduced the number of rebound bursts (before Z944: 1.2 ± 0.2, after Z944: 0.0 ± 0.0; *t*_(4)_ = 6.0, *p *=* *0.004, *n* = 5) and rebound AP frequency (before Z944: 49.2 ± 15.5 Hz, after Z944: 5.6 ± 3.6 Hz; *t*_(5)_ = 3.0, *p *=* *0.029, *n* = 6; Fig. 8*D–F*). Thus, T-type channel activation contributes to both depolarization-induced burst firing from a hyperpolarized *V*_m_ and rebound burst firing.

A previous study found that NMDA and AMPA receptor activation contributes to burst firing in the LHb (Yang et al., 2018). In MHb neurons which exhibited ramp depolarization-induced burst firing from a *V*_m_ <−60 mV, the NMDA receptor antagonists CPP (5 μm) or AP-5 (50 μm) were bath-perfused. Neither CPP nor AP-5 significantly affected the number of bursts per ramp (before CPP: 6.0 ± 1.2, after CPP: 6.8 ± 1.1; *t*_(3)_ = −1.42, *p *=* *0.25, *n* = 4; before AP-5: 3.2 ± 0.7, after AP-5: 2.4 ± 0.4; *t*_(4)_ = 1.4, *p *=* *0.24, *n* = 5) or the average number of APs per burst (before CPP: 2.3 ± 0.2, after CPP: 2.2 ± 0.1; *t*_(3)_ = 0.65, *p *=* *0.57, *n* = 4; before AP-5: 2.1 ± 0.1, after AP-5: 2.4 ± 0.2; *t*_(4)_ = −2.3, *p *=* *0.08, *n* = 5). Similarly, the AMPA receptor antagonist CNQX did not significantly affect the number of bursts per ramp (before CNQX: 3.2 ± 0.7, after CNQX: 2.6 ± 0.5; *t*_(4)_ = 2.5, *p *=* *0.07, *n* = 5) or the average number of APs per burst (before CNQX: 2.1 ± 0.1, after CNQX: 2.1 ± 0.1; *t*_(4)_ = 0.02, *p *=* *0.99, *n* = 5). Therefore, tonic synaptic glutamate does not contribute to depolarization-induced burst firing from a hyperpolarized *V*_m_ in MHb neurons in brain slices.

### Voltage-gated Ca^2+^ currents in MHb neurons

**Figure 9.**
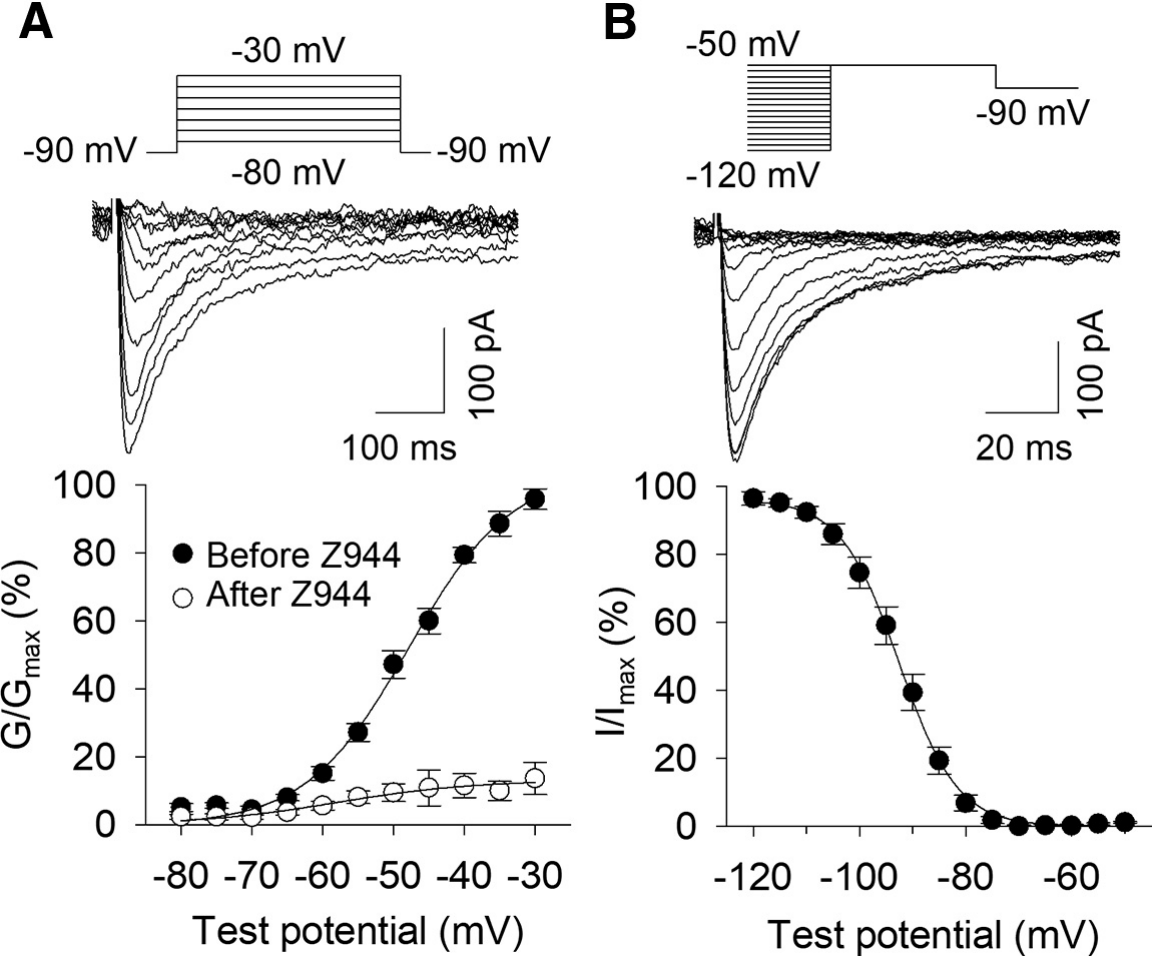
Voltage-gated Ca^2+^ currents in MHb neurons. ***A***, top, Representative currents induced by step depolarization from −90 mV to progressively more depolarized test potentials in the absence of Z944. Bottom, Voltage dependence of activation for voltage-gated Ca^2+^ currents before and after Z944 perfusion. ***B***, top, Representative currents evoked by step depolarization to −50 mV after different initial test potentials. Bottom, Voltage dependence of inactivation for voltage-gated Ca^2+^ currents.

Next, we tested whether voltage-gated Ca^2+^ currents could be evoked in MHb neurons, and we determined the voltage dependence of their activation and inactivation. Ca^2+^ currents were recorded similarly to previous studies (Joksimovic et al., 2017; Stamenic and Todorovic, 2018), using an internal solution that caused rapid rundown of high-voltage activated (HVA) Ca^2+^ currents (Todorovic and Lingle, 1998). Ca^2+^ current activation was studied using a step depolarization protocol where MHb neurons were stepped to progressively more depolarized holding potentials for 320 ms after initially holding neurons at −90 mV for 1 s (Fig. 9*A*). Rapid inward currents were evoked, and the conductance versus voltage relationship was fit with a Boltzmann function. The average voltage of half-activation (*V*_50_) in the absence of Z944 was −47.9 mV. There was a significant effect of Z944 to reduce Ca^2+^ current amplitude (two-way ANOVA with repeated measures: *F*_(1,79)_ = 246.4, *p *<* *0.001; Holm–Sidak *post hoc* test: *p *<* *0.001). Thus, Ca^2+^ currents can be evoked in MHb neurons and are sensitive to T-type channel blockade.

The voltage dependence of Ca^2+^ current inactivation was determined by holding MHb neurons for 3.6 s at progressively more depolarized membrane potentials before a 320-ms step to −50 mV (Fig. 9*B*). Rapid inward currents were evoked when neurons were pre-held at hyperpolarized membrane potentials but were not evoked when pre-held at more depolarized potentials. A plot of the current versus initial holding voltage was fit with a Boltzmann function. The average voltage of half-inactivation (*V*_50_) was −92.6 mV. These voltages of half-activation and half-inactivation are consistent with those measured for T-type channels in the rat subiculum using an identical internal solution and voltage protocols (Joksimovic et al., 2017). Collectively, these results indicate that evoked Ca^2+^ currents in MHb neurons are likely to be mediated by T-type channels.

### Depolarized AP doublets

**Figure 10.**
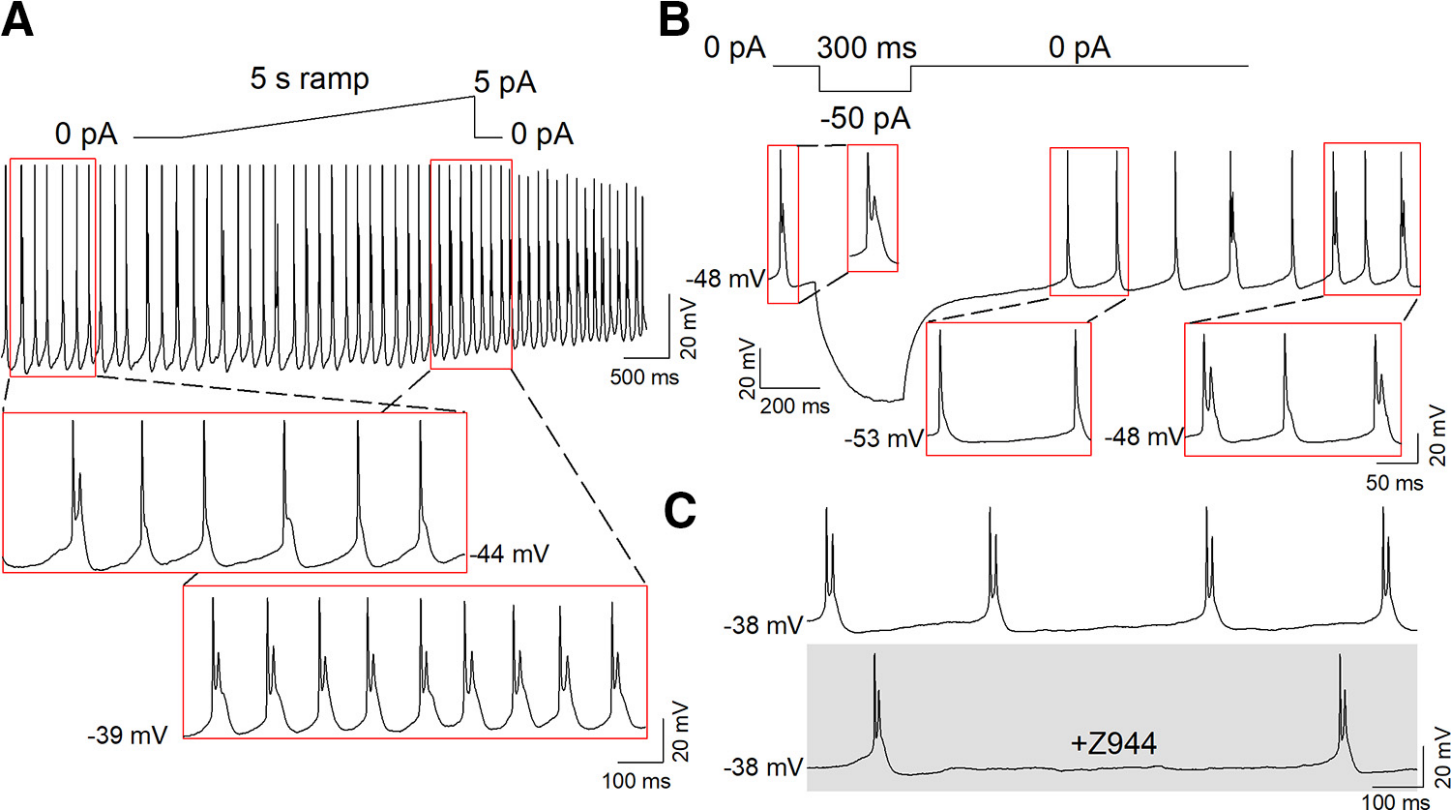
AP doublet firing at depolarized membrane potentials is not T-type channel mediated. ***A***, AP doublet firing at depolarized membrane potentials could be enhanced by low-level ramp depolarization. ***B***, Hyperpolarization caused a transition to tonic firing in depolarized AP doublet firing neurons, which returned to AP doublet firing on subsequent spontaneous depolarization. ***C***, Depolarized AP doublet firing was not blocked by perfusion of Z944.

Eight of 130 total neurons (6.2%) spontaneously fired AP doublets and had an average *V*_m_ of −43.9 ± 1.5 mV (Fig. 1*D*). As T-type channels are likely completely inactivated at this *V*_m_, we aimed to further characterize the electrophysiological characteristics of depolarized AP doublet firing. In a depolarized (*V*_m_ = −44 mV) neuron displaying both spontaneous AP doublets and single spikes with a large afterdepolarization, low-level ramp depolarization (5 pA, 5 s) increased AP doublet firing at a *V*_m_ of −39 mV (Fig. 10*A*). In a different MHb neuron that spontaneously fired AP doublets at a *V*_m_ of −48 mV, membrane hyperpolarization (−50 pA, 300 ms) briefly converted depolarized AP doublet firing to tonic firing at a *V*_m_ of −53 mV, followed by a return to AP doublet firing with spontaneous depolarization to a *V*_m_ of −48 mV (Fig. 10*B*). Spontaneous AP doublets at depolarized membrane potentials were not blocked by perfusion with Z944 (Fig. 10*C*; *n* = 5). Thus, AP doublet firing at sustained depolarized potentials does not require T-type channel activation.

## Discussion

In this study, we found that a subpopulation of MHb neurons are capable of firing high-frequency AP bursts from a hyperpolarized *V*_m_ that strongly resemble burst firing mediated by T-type Ca^2+^ channel activation (Llinás and Jahnsen, 1982; McCormick and Pape, 1990; Cheong and Shin, 2013; Lambert et al., 2014). This burst firing could be induced by rebounding from membrane hyperpolarization or depolarization from a hyperpolarized *V*_m_, whereas depolarization from a *V*_m_ > −60 mV led to tonic AP firing or depolarized AP doublet firing. Using RNAscope *in situ* hybridization, we identified robust mRNA expression of the T-type Ca^2+^ channel Ca_v_3.1 primarily in the lateral MHb, where it co-localized with both ChAT and Tac1, markers which define the ventral and dorsal MHb, respectively (Molas et al., 2017). Expression of Ca_v_3.2 and Ca_v_3.3 was also observed in the MHb but was comparatively sparse. Hyperpolarization-induced and depolarization-induced burst firing from a hyperpolarized *V*_m_, and evoked Ca^2+^ currents, were blocked by bath perfusion of the selective Ca_v_3 antagonist Z944. Thus, a subpopulation of MHb neurons are capable of high-frequency burst firing, in contrast to previous reports of exclusive tonic firing by MHb neurons, with burst firing from a hyperpolarized *V*_m_ sensitive to T-type channel blockade.

Interestingly, single-cell RNA sequencing of the mouse habenula revealed that transcriptomically defined clusters of MHb neurons heterogeneously express Ca_v_3.1 (Hashikawa et al., 2019). The highest Ca_v_3.1 expression was observed in neuron clusters located in the lateral aspect of both the dorsal and ventral MHb and in clusters which express Tac1 or ChAT, consistent with our results. This study also identified mRNA for Ca_v_3.3 in some MHb clusters. A previous autoradiographic ISH study concluded that mRNA for Ca_v_3.1 was expressed in the LHb but not the MHb (Talley et al., 1999), and hyperpolarization-induced rebound burst firing mediated by T-type channels has been observed in the LHb (Cui et al., 2018; Yang et al., 2018).

Voltage-gated Ca^2+^ channels fall into two broad categories: HVA and LVA channels (Perez-Reyes, 2003; Cheong and Shin, 2013). T-type Ca^2+^ channels are LVA channels, whereas the other families of voltage-gated Ca^2+^ channels, including L-type, P/Q-type, N-type, and R-type channels, are HVA channels. Unique properties of T-type Ca^2+^ channels differentiate them from the HVA family. In contrast to HVA channels, T-type Ca^2+^ channels require less membrane depolarization to open and rapidly inactivate with prolonged depolarization, leading to low-threshold Ca^2+^ spikes (LTS) that can trigger a burst of Na^+^ and K^+^-mediated APs on top of the Ca^2+^-mediated LTS. In neurons with a resting membrane potential of −60 mV, the majority of T-type Ca^2+^ channels are inactivated, such that membrane hyperpolarization alleviates inactivation and leads to rebound burst firing on subsequent depolarization (Perez-Reyes, 2003; Cheong and Shin, 2013). In more hyperpolarized neurons, depolarization can trigger burst firing, but sustained and/or stronger depolarization inactivates T-type Ca^2+^ channels and prevents burst firing (Perez-Reyes, 2003; Cheong and Shin, 2013).

We observed that ∼20% of MHb neurons burst fired to ramp depolarization from a hyperpolarized *V*_m_ or displayed rebound burst firing. This bursting was observed in neurons that originally displayed all types of activity states at baseline, indicating that although T-type channel-mediated bursting is rare at resting conditions, a larger population of MHb neurons are capable of burst firing. Interestingly, the LHb similarly has a low percentage of spontaneously bursting neurons at baseline in Sprague Dawley rats and C57BL/6 mice, whereas congenitally learned helpless rats and mice exposed to chronic restraint stress have a substantially greater percentage of LHb neurons that burst at baseline (Yang et al., 2018). It is possible that the percentage of MHb neurons displaying different activity states may be altered in behavioral states regulated by the MHb, such as elevated anxiety-like states or nicotine withdrawal.

We also found that some neurons burst fired during ramp depolarization but did not exhibit rebound bursting following 100-ms or 1.5-s hyperpolarization. This is likely because depolarization following hyperpolarization is necessary for T-type Ca^2+^ channel activation. Hyperpolarization-activated cyclic nucleotide-gated (HCN) channels, in particular HCN3 and HCN4, are expressed in the MHb in rodents (Monteggia et al., 2000; Santoro et al., 2000; Notomi and Shigemoto, 2004; Görlich et al., 2013). Consistent with this, depolarizing current sag was frequently observed with 1.5-s hyperpolarization (Fig. 3*D*), which is a hallmark of HCN channel activation (Biel et al., 2009). It is thus likely that HCN channel activation can provide the rebound depolarization necessary to activate T-type Ca^2+^ channels in some MHb neurons.

In addition to burst firing from a hyperpolarized *V*_m_, some neurons displayed high-frequency AP doublet firing at depolarized potentials (average *V*_m_ = −45.2 ± 1.5 mV). This AP doublet firing was not affected by T-type channel blockade. This is unsurprising, as T-type channels are likely completely inactivated at these depolarized potentials. The ionic conductances mediating this depolarized burst firing remain unknown. In neurons in other brain regions, persistent Na^+^ current can contribute to afterdepolarization and promote burst firing (Azouz et al., 1996; Brumberg et al., 2000; Su et al., 2001). It is possible that persistent Na^+^ current or other ionic conductances mediate this depolarized burst firing.

In summary, we demonstrated that a subpopulation of MHb neurons are capable of high-frequency AP firing, in contrast to previous reports that MHb neurons solely fire tonic APs at a frequency of ∼2–10 Hz. Two distinct modes of high-frequency AP firing were observed, including T-type channel-mediated bursting and T-type channel-independent AP doublets. T-type channel-mediated bursting occurred from a previously hyperpolarized *V*_m_, whereas T-type channel-independent bursting remained with sustained depolarization and was not blocked by Z944. Ca_v_3 mRNA was expressed in the MHb, most notably Ca_v_3.1 in the lateral MHb, consistent with the predominant location of bursting MHb neurons. Thus, T-type Ca^2+^ channels may contribute to the regulation of behaviors relevant to neuropsychiatric disease by shaping the frequency and pattern of MHb neuron AP firing.

## Synthesis

Reviewing Editor: Pablo Castillo, Albert Einstein College of Medicine Decisions are customarily a result of the Reviewing Editor and the peer reviewers coming together and discussing their recommendations until a consensus is reached. When revisions are invited, a fact-based synthesis statement explaining their decision and outlining what is needed to prepare a revision will be listed below. The following reviewer(s) agreed to reveal their identity: Hailan Hu.

Your manuscript has been evaluated by two experts in the field. Both agree on the significance of your findings and important contribution of your study. However, as you will see below, they also raised some comments that you should address in the revision.

Editor’s note: summary plots (bar graphs: figs. 1G, 5B,C) should include individual measurements.

REVIEWER 1

In this manuscript, the authors characterized the firing patterns of MHb neurons using slice electrophysiology. In contrast to previous reports showing solely tonic firing, the authors identified five firing patterns in the MHb. They then focused on the bursting characteristic. Although only ∼5% MHb neurons display burst firing at the baseline, through ramp-up current injection combined with hyperpolarization or depolarization to move membrane potential into the right range for T-VSCC deinactivation, the authors were able to induce burst firing in 20% MHb neurons. These bursts can be blocked by a T-VSCC antagonist Z944. RNAscope confirmed expression of T-VSCC, especially Cav3.1, in the MHb. Collectively, this study provides valuable information for future studies on MHb function. I have just a few questions for authors to clarify before its warranty for publication.

1.Since depolarization currents through AMPA receptors under the physiological condition may provide the drive to activate T-VSCC, they authors should try to exclude CNQX from their recording solution to see whether the percentahge of neurons capable of firing bursts may be even higher.

2.The burst firing in the neighboring LHb was found to depend on both T-VSCCs and NMDA receptors. The authors should test whether NMDA receptors are also required for the MHb bursts.

3.The authors noted that “Of the 26 neurons where location information was recorded, 18 of 26 (69.2%) of these neurons were in the lateral or central MHb”. It would be nice to show a schematic map indicating the locations of different types of neurons.

REVIEWER 2

This study characterizes a low-voltage-activated T-type Ca2+ in mediating a unique mode of neuronal firing in medial habenular neurons. The studies are systematically designed and carefully carried out, and the data quality is high. The narrative is clear, and the conclusions are well supported by the results. This carefully characterized firing patterns of MHb neurons and the underlying ionic mechanisms are important for us to better understand the neural encoding by MHb neurons under physiological and pathological conditions. One minor concern is listed below.

Fig. 5: Cav3.1 mRNA expression density in the MHb is shown to be very different between 5B and 5C. This needs to be clarified.

## Author Response

We thank the reviewers for their time and effort in critiquing this manuscript. Please find below our point-to-point responses to the critiques raised. New edits in the manuscript have been labeled with blue font.

REVIEWER 1

In this manuscript, the authors characterized the firing patterns of MHb neurons using slice electrophysiology. In contrast to previous reports showing solely tonic firing, the authors identified five firing patterns in the MHb. They then focused on the bursting characteristic. Although only ∼5% MHb neurons display burst firing at the baseline, through ramp-up current injection combined with hyperpolarization or depolarization to move membrane potential into the right range for T-VSCC deinactivation, the authors were able to induce burst firing in 20% MHb neurons. These bursts can be blocked by a T-VSCC antagonist Z944. RNAscope confirmed expression of T-VSCC, especially Cav3.1, in the MHb. Collectively, this study provides valuable information for future studies on MHb function. I have just a few questions for authors to clarify before its warranty for publication.

1.Since depolarization currents through AMPA receptors under the physiological condition may provide the drive to activate T-VSCC, they authors should try to exclude CNQX from their recording solution to see whether the percentage of neurons capable of firing bursts may be even higher.

We directly tested whether AMPA receptor activation affects burst firing in MHb neurons by perfusing CNQX onto MHb neurons that bursted on ramp depolarization from a membrane potential < -60 mV. With this before-and-after comparison from the same cell, we found that CNQX did not significantly affect the number of bursts per ramp or the average number of APs per burst. MHb neurons display sparse spontaneous AMPAR-EPSCs (averaged at ∼0.2 Hz) with small amplitude (mean = 8 pA) in adult mouse slices (PMID: 27793697). We confirmed this finding in our slices. In addition, high-intensity electrical stimulation evoked small or no EPSCs in MHb neurons. Thus, glutamate input to MHb neurons in slice recordings is very weak. It is likely that excitatory inputs do not play a significant role in driving burst firing in MHb neurons, particularly in brain slice conditions.

2.The burst firing in the neighboring LHb was found to depend on both T-VSCCs and NMDA receptors. The authors should test whether NMDA receptors are also required for the MHb bursts.

We have now tested the effect of NMDA receptor blockade on burst firing in MHb neurons. We tested whether perfusion of the NMDAR antagonist CPP (5 μM) or AP-5 (50 μM) affected burst firing to ramp depolarization from a hyperpolarized Vm. This before-and-after comparison revealed that NMDAR blockade did not significantly affect the number of bursts per ramp or the average number of APs per burst. This lack of NMDAR requirement differs from that observed in the LHb. However, as mentioned above, tonic glutamate input to MHb neurons in slice recordings is weak. This likely explains why burst firing in MHb neurons is not affected by tonic NMDAR activation.

3.The authors noted that “Of the 26 neurons where location information was recorded, 18 of 26 (69.2%) of these neurons were in the lateral or central MHb”. It would be nice to show a schematic map indicating the locations of different types of neurons.

We have now provided a map indicating the locations of these bursting neurons in Fig. 6. We also added new bursting neurons from our most recent recordings and now indicate that of the 35 neurons where location information was recorded, 27 of 35 (77%) were in the lateral or central MHb.

REVIEWER 2

This study characterizes a low-voltage-activated T-type Ca2+ in mediating a unique mode of neuronal firing in medial habenular neurons. The studies are systematically designed and carefully carried out, and the data quality is high. The narrative is clear, and the conclusions are well supported by the results. This carefully characterized firing patterns of MHb neurons and the underlying ionic mechanisms are important for us to better understand the neural encoding by MHb neurons under physiological and pathological conditions. One minor concern is listed below.

Fig. 5: Cav3.1 mRNA expression density in the MHb is shown to be very different between 5B and 5C. This needs to be clarified.

Cav3.1 mRNA expression density in Fig. 5B reflects its expression in the entire MHb, containing both the medial and lateral regions. Fig. 5C shows the expression of Cav3.1 in each region of the MHb separately. The average expression density of Cav3.1 in the lateral MHb is 0.011 puncta/μm2, whereas its average expression density in the medial MHb is 0.002 puncta/μm2. The expression density in the entire MHb (0.066 puncta/μm2) is essentially equal to the mean of these expression densities (0.0065 puncta/μm2).
